# How intra-familial decision-making affects women’s access to, and use of maternal healthcare services in Ghana: a qualitative study

**DOI:** 10.1186/s12884-015-0590-4

**Published:** 2015-08-15

**Authors:** John Kuumuori Ganle, Bernard Obeng, Alexander Yao Segbefia, Vitalis Mwinyuri, Joseph Yaw Yeboah, Leonard Baatiema

**Affiliations:** Department of Population, Family and Reproductive Health, School of Public Health, University of Ghana, Accra, Ghana; Department of Sociology & Social Work, Faculty of Social Sciences, Kwame Nkrumah University of Science & Technology, Kumasi, Ghana; Population, Health and Gender Research Group, Department of Geography and Rural Development, Kwame Nkrumah University of Science and Technology, Kumasi, Ghana

## Abstract

**Background:**

There is some evidence to suggest that within the household, family and community settings, women in sub-Saharan Africa often have limited autonomy and control over their reproductive health decisions. However, there are few studies that examine how intra-familial decision-making power may affect women’s ability to access and use maternal health services. The purpose of this paper is to examine how intra-familial decision-making affects women’s ability to access and use maternal health services.

**Methods:**

We conducted 12 focus group discussions and 81 individual interviews with a total of 185 expectant and lactating mothers in six communities in Ghana. In addition, 20 key informant interviews were completed with healthcare providers. Attride-Stirling’s thematic network analysis framework was used to analyse the data.

**Results:**

Findings suggest that decision-making regarding access to and use of skilled maternal healthcare services is strongly influenced by the values and opinions of husbands, mothers-in-law, traditional birth attendants and other family and community members, more than those of individual childbearing women. In 49.2 %, 16.2 %, and 12.4 % of cases in which women said they were unable to access maternal health services during their last pregnancy, husbands, mothers-in-law, and husband plus mothers-in-law, respectively, made the decision. Women themselves were the final decision-makers in only 2.7 % of the cases. The findings highlight how the goal of improving access to maternal healthcare services can be undermined by women’s lack of decision-making autonomy through complex processes of gender inequality, economic marginalisation, communal decision-making and social power.

**Conclusion:**

Interventions to improve women’s use of maternity services should move beyond individual women to target different stakeholders at multiple levels, including husbands and mothers-in-law.

## Background

Much progress has been made over the last several decades to improve maternal health worldwide. This progress notwithstanding, high maternal mortality persists in many resource-poor settings particularly in sub-Saharan Africa [1]. While facility births have gone up dramatically in some settings, some women still do not have access to health facilities and skilled birth attendants in many countries in the sub-Saharan African region where the burden of maternal mortality is relatively high [2–6]. For instance while in East Asia and the Pacific as well as in Latin America and the Caribbean, about 9 in 10 births occur in health facilities with a skilled birth attendant, in sub-Saharan Africa only about half of births (46 %) are delivered in a health facility with a skilled birth attendant [7].

Like many countries in sub-Saharan Africa, Ghana has had a persistently high maternal mortality rate [8]. According to the WHO’s most recent estimates, Ghana’s maternal mortality ratio stands at 380 per 100,000 live births [7]. Maternal mortality accounts for 14 % of deaths among females aged 12–49 years, and is the second largest cause of female mortality after infectious diseases among women of childbearing age [9]. Despite the fact that Ghana has since 2003 implemented a maternal healthcare policy that provides free maternity care services in all public and mission healthcare facilities, in parts of Ghana more than 45 % of births still occur at home without any form of skilled care [10]. In addition, large and growing gradients of inequalities in skilled care services accessibility and utilisation have been observed in Ghana [2, 6, 8]. Two recent World Bank studies have suggested that Ghana is off track to achieving the fifth Millennium Development Goal (MDG 5) of three-quarters reduction in maternal mortality ratio between 1990 and 2015 [11, 12]. According to one of the studies, among countries with similar levels of income and health expenditure, Ghana performed worse than average with respect to neonatal, infant, under-five, and maternal mortality [11].

At both global and national levels, there have been different explanations for the persistence of poor maternal health. Limited access to preventive and curative services [13], inadequate health infrastructure and personnel [13, 14], and inability to pay [13], have been recognised as formidable barriers to receipt of maternal healthcare. But recent research indicate that supply side factors alone do not fully explain the variability in women’s ability to seek care as substantial gaps in coverage and other socio-cultural barriers remain even after adjustment for the availability of services [15, 16]. In Ghana for example, a number of recent studies suggest that social factors like religious beliefs, cultural norms concerning pregnancy management and the need to seek permission from husbands and compound heads before care is accessed play important roles in determining whether women deliver at home or in a health facility [4, 5, 17, 18].

Consequently, programme planners and researchers have begun to appreciate the complexity of contextual influences on maternal healthcare seeking behaviours, and have accordingly adopted an approach that recognises individual women’s attitudes and behaviours as products of their social and cultural environments [19, 20]. Even then, most studies have focused on the knowledge, attitudes, and practices of women as a target population [20]. However, this narrow focus is incongruent with sociocultural contexts in which women hold low social and economic status, and where women’s healthcare decision-making abilities are subject to other factors such as power dynamics within the household [21, 22]. There is some evidence to suggest that within the household, family and community settings, women in sub-Saharan Africa often have limited autonomy and control over their reproductive health decisions, and this often results in sub-optimal use of skilled maternal health services [23–26]. Indeed, a number of empirical studies in varying contexts have examined the role of women’s autonomy and decision-making power in facilitating access to skilled maternity care [21–27]. In sub-Saharan Africa more specifically, increased female autonomy has been linked to improved utilisation of maternal health services [19, 21, 25, 26], and studies in several sub-Saharan African countries including Ethiopia, Gambia, Mali, Nigeria, Swaziland, Tanzania and Tunisia all indicate that women of childbearing age often do not decide whether or not to seek healthcare [17, 21, 26, 28]. In the context of Ghana, few recent studies have also begun to examine decision-making around maternal health seeking. For instance research by Bazzano et al. [29], Mills and Bertrand [30], Moyer et al. [17], and Gupta et al. [18] suggest that rather than women making decisions to access or use maternal health services by themselves, these decisions are often made on their behalf by their spouse or a senior member of their family such as their mother-in-law, father-in-law, grandmother, or compound head. Another study on female autonomy and reported abortion-seeking in Ghana found that with increasing autonomy, women are more likely to report the termination of pregnancy [31]. While these studies have provided important insights into how social factors can influence women’s ability to access skilled care, few of them have extended their scope beyond rural northern Ghana where families are patrilineal and the eldest male or compound head, typically has the final say in all decisions [17]. Given that many societies in southern Ghana are matrilineal, it is not clear whether the same patterns of decision-making exist. Extending this growing body of research to cover other Ghanaian contexts will therefore be important not only for understanding of how intra-familial decision-making influences women’s access to maternal health, but also for instituting the needed remedial policy actions to address the situation. The aim of this paper is to address this gap by exploring how intra-familial decision-making within the household, family and community affects women’s ability to access and use maternal health services in Ghana, focusing on communities in both southern and northern Ghana. The findings of this paper contribute to the growing body of empirical research on the influences of women’s autonomy and intra-familial decision-making on maternal health seeking behaviour in Ghana.

## Conceptual background

Women’s ability to make decisions either alone or in consultation with other people at the level of the family or household is an important determinant of their access to, and use of, skilled maternal healthcare services. Within the literature related to maternal health, women’s decision-making ability as regards access to, and use of, health services is often discussed using the concept of autonomy [25–27]. Autonomy is a multidimensional concept and there is a range of philosophical theories and debates about what autonomy is and how it should be understood [32, 33]. Notwithstanding the complexities of these philosophical debates, autonomy may be viewed as the idea that people should be able to reflect on their own best interests and make decisions for themselves. In the words of Beauchamp and Childress [33].

‘Personal autonomy encompasses, at minimum, self-rule that is free from both controlling interference by others and from certain limitations such as inadequate understanding that prevents meaningful choice. The autonomous individual acts freely in accordance with a self-chosen plan…A person of diminished autonomy, by contrast, is in some respect controlled by others or incapable of deliberating or acting on the basis of his or her desires and plans’.

In the specific case of women’s autonomy, it means that a woman has the capacity and freedom to act (e.g. the ability to go to a health facility alone or without asking anyone’s permission) independently. The essential conditions for autonomy in this context, and indeed which most theories on the concept acknowledge, are *liberty* (independence from controlling influences) and *agency/self-efficacy* (capacity for intentional action) [33].

In the fields of healthcare and health services research, the concept of autonomy has recently been operationalised and attempts made to evaluate its effects on for example, women’s ability to access maternal and reproductive health services [21, 25–27, 31]. It is within this context that the present paper aims to contribute to the understanding of how women’s autonomy within the household, family and community affect their ability to access and use skilled maternal healthcare services in Ghana.

In earlier studies, women’s education and employment served as the most frequently used proxies of women’s autonomy [32, 34]. More recently however, scholars have moved away from using these proxies to choosing instead, more direct aspects [26]. These direct aspects of autonomy consist of a combination of observable indicators that tap into different dimensions of the concept, including freedom of mobility, ability to make decisions or to participate in childbirth related household decision-making, and self-efficacy [31, 35, 36]. In consonance with these contemporary studies, women’s participation in decision-making, freedom of mobility as well as self-efficacy regarding access to and use of maternity care services were used as indicators of autonomy in our study.

Previous research in low-income settings suggest that women’s active participation in domestic decision-making, freedom of movement and self-efficacy are a reflection of their power and autonomy within the household, and might increase their chances of making the right reproductive choices including accessing and utilising maternal health services [26, 37]. Women’s participation in intra-familial decision-making can particularly enable them to influence a greater number of strategic decisions, including decisions to go to the hospital to seek skilled birthing care. In many Ghanaian communities where men’s domination over women is strongest within the household, women’s ability to participate and influence decisions that affect their lives at the household level can be one of the principal components of their autonomy and empowerment. With regard to women’s freedom of mobility, Fernardo and Porter have argued that increasing women’s mobility can empower them to exercise greater control over their lives by increasing their access to healthcare, education, markets and information [38]. A study in India has shown that women with greater freedom of movement are more likely to access antenatal and delivery care [24].

Rather than following the quantitative approach that some previous studies adopted [21, 24–27], a qualitative approach was used in this study, which allowed for an in-depth understanding of the processes through which women’s autonomy affected their ability to access maternity care services. The lack of this kind of depth of understanding is a fundamental weakness of previous studies that adopted a quantitative approach to the evaluation of the role of women’s autonomy in maternal healthcare access [16].

## Methods

### Research design

The qualitative data reported in this paper were extracted from within a larger, original study that was conducted to examine the effects of Ghana’s free maternal healthcare policy on women’s maternity care seeking experience, equity of access, and barriers to accessibility and utilization of maternal and newborn healthcare services [2–6]. The design of this larger study was mixed – it involved analysis of a nationally representative retrospective household survey data in combination with qualitative exploration using focus group discussions (FGDs), individual interviews, (IIs), key informant interviews (KIIs), case studies and structured field observations. The focus in this paper is on reporting findings from the qualitative component of this larger study, which examined how intra-familial decision-making affected women’s ability to access and use maternal health services. For details on the quantitative component of study, see [2, 6].

### Study context

Empirical research was conducted between November 2011 and May 2012 in a total of 6 purposively sampled communities, namely Kuntanase, Abono, and Piase in the Bosomtwe district of the Ashanti region; and Mpaha, Sankpala and Tidrope in the Central Gonja district of the Northern region of Ghana. Ghana is a West African country, covering a total land area of 238,305 square kilometres. Tables 1 and 2 give a summary of selected demographic, socio-economic, and health indicators of Ghana and the 6 study communities.

**Table 1:** Selected demographic, socio-economic, and health indicators of Ghana

Indicator	Statistic	Source of Statistic
Total population (2010)	24,658823 (48.8 % male; 51.2 % female)	Ghana 2010 Population and Housing Census
Proportion of population below 15 years (%) (2010)	38.3	Ghana 2010 Population and Housing Census
Proportion of population above 60 years (%) (2010)	4.7	Ghana 2010 Population and Housing Census
Proportion of population living in urban areas (%) (2010)	50.9	Ghana 2010 Population and Housing Census
Adult literacy rate (%) (2008)	57.9	Ghana Demographic & Health Survey 2008
Percentage of Population with No Education (2008)	13.3 (male); 21.2 (female)	Ghana Demographic & Health Survey 2008
Percentage of Population with No Education (Rural) (2008)	19.9 (male); 30.8 (female)	Ghana Demographic & Health Survey 2008
Percentage of Population with No Education (Urban) (2008)	5.6 (male); 10.9 (female)	Ghana Demographic & Health Survey 2008
Total gross domestic product (US$ in billion) (2009)	34.0	Schieber *et al*. [10]
Gross national income (GNI) per capita (US$) (2009)	700	Schieber *et al*. [10]
Annual economic growth rate (%) (2009)	6.3	Schieber *et al*. [10]
Population living on less than 1 US$ dollar per day (2005)	30	Schieber *et al*. [10]
Multi-dimensional poverty Index (2008)	0.14	Schieber *et al*. [10]
Human development Index (2009)	0.526	Schieber *et al*. [10]
Adult mortality rate per 1,000 population (2007)	343 (male); 291 (female)	World Health Statistics 2009
Life expectancy at birth (in years) (2009)	59 (male); 60.7 (female)	World Health Statistics 2009
Maternal mortality ratio (2014)	380 per 100,000 live births	World Health Organisation [7]
Infant Mortality Rate (per 1,000 live births) (2008)	50	World Health Statistics 2009
Under 5 Mortality Rate (per 1,000 live births) (2008)	80	World Health Statistics 2009
Neonatal Mortality Rate (per 1,000 live births) (2008)	30	World Health Statistics 2009
Post-Neonatal Mortality Rate (per 1,000 live births) (2008)	21	World Health Statistics 2009
Crude Birth Rate (per 1,000) (2008)	29	World Health Statistics 2009
Crude Death Rate (per 1,000) (2008)	9.4	World Health Statistics 2009
Total Fertility Rate (2008)	4	World Health Statistics 2009

**Table 2:** Characteristics of the study communities

	Bosomtwe District				Central Gonja District			
Community Characteristic	Piase	Abono	Kuntanase	Entire Bosomtwe District	Tidrope	Mpaha	Sankpala	Entire Central Gonja District
Population (2010)	2,772	1,467	34.682	99,964	1,025	4,126	1,526	110,576
Number and type of health facility (2010)	Health centre (1)	0	Hospital (1)	Hospitals (3), Health centres (3), Clinics (7), Maternity Homes (3)	None	Health centre (1)	Health centre (1)	Hospital (1), Health centres (5) CHPS zones (11)
Number of medical doctors (2010)	0	0	2	10	0	0	0	1
Number of midwives (2010)	1	0	3	38	0	0	1	9
Number of general nurses (2010)	0	0	7	65	0	0	0	6
Number of community Health nurses (2010)	2	0	11	25	0	5	4	37
Number of pharmacists (2010)	0	0	1	3	0	0	0	0
Number of dispensary technicians (2010)	0	0	3	14	0	1	0	2
Distance to nearest health facility (km)	1	12	1	n/a	13	2	1	n/a
Doctor to patient ratio (2010)	1: 9,997				1: 110,576			
Nurse to patient ratio (2010)	1: 1,111				1:2,572			
Number of maternal deaths (2010)	n/a	n/a	n/a	5	n/a	2	0	28
Number of under-5 deaths (2010)	n/a	n/a	n/a	25	n/a	10	4	25
Skilled Delivery (%) (2010)	56	n/a	74	54.3	n/a	18.1	22.2	14.4

Preventive and curative healthcare services delivery in Ghana is generally decentralised, and the healthcare system is organised under four main categories of delivery systems: public, private-for-profit, private-not-for-profit, and traditional systems [39]. Community-based Health Planning and Services (CHPS) compounds, health centres, mission clinics and private midwifery homes provide basic obstetric and antenatal care services in most communities. Each health centre serves a population of approximately 20,000 [8]. In most rural areas, untrained traditional birth attendants (TBAs) carry out deliveries. Comprehensive skilled emergency obstetric care is mostly available from urban district and regional hospitals, as well as national referral hospitals. But some private hospitals also provide comprehensive care. The Ghana Health Service runs most hospitals though the mission sector plays a significant role, especially in more remote regions. The payment mechanism for healthcare is a combination of fee-exemption, health insurance and out-of-pocket [11, 12].

As noted earlier, many societies in Ghana, particularly societies in northern Ghana, are patriarchal. Women in Ghana are also still generally more disadvantaged - educational and economic opportunities for women are limited - from a young age compared to men [4, 5]. These socio-economic and cultural contexts make Ghana an ideal case for investigating how intra-familial decision-making influences women’s maternal health-seeking behaviours. The six communities were chosen not only to capture a divide between a relatively destitute and patriarchal northern Ghana and a relatively prosperous and matrilineal southern Ghana, but also to provide a diversity of social and health situations (see Table 2). For example, Kuntanase and Mpaha were selected to represent urban communities in the Bosomtwe and Central Gonja districts respectively; Piase and Sankpala were chosen to represent rural communities with health facilities in the Bosomtwe and Central Gonja districts respectively; while Abono and Tidrope were selected to represent rural communities without any health facilities in the Bosomtwe and Central Gonja districts respectively. These differences should allow for some comparative analysis.

### Research participants

The research participants comprised pregnant women and lactating mothers, and healthcare providers. The women consisted of those who were pregnant at the time of this research or had given birth between January 2011 and May 2012. Table 3 shows the socio-demographic characteristics of women participants.

**Table 3:** Socio-demographic characteristics of women participants (N = 185)

Characteristic	Number of Women	Percentage
Age (yrs)		
≥20	42	22.7
21-25	51	27.6
26-30	50	27
31-35	18	9.7
36-40	7	3.8
41-45	2	1.1
Don’t know	15	8.1
Highest Level of Education		
None	112	60.7
Primary	38	20.4
Middle/JSS	32	17.3
Secondary+	3	1.6
Occupation		
Farming	74	40
Petty trading	42	22.7
Unemployed	42	22.7
Hair dresser	12	6.5
Seamstress	13	7
Teacher	2	1.1
Marital Status		
Married	128	69.1
Widow	9	4.9
Separated	17	9.2
Single	31	16.8
Religion		
Christian	91	49.2
Muslim	78	42.2
Traditional African Religion	14	7.6
Other	2	1
Type of Marriage		
Monogamous	102	54.7
Polygynous	83	45.3
Community		
Kuntanase	35	19
Abono	28	15
Piase	35	19
Mpaha	29	16
Sankpala	32	17
Tidrope	26	14
Residence		
Rural	120	65
Urban	65	35
Number of Children		
None	17	9.2
1-3	99	53.5
4-6	69	37.3
Total Number of Pregnancies Ever Had		
1-3	106	57.3
4-6	65	35.1
7-9	14	7.6
Age at First Pregnancy		
15-20	71	38.4
21-30	99	53.5
Don’t know	15	8.1
Place of Last Delivery		
Home	117	63.3
Health Facility	68	36.7

The majority of the participants (65 %) were rural women. The ages of these women varied between 18 and 45 years. The majority of the women (60.7 %) had no formal education. A few of the women were unemployed while most were engaged in diverse self-employed activities such as farming, trading, hairdressing, dressmaking, and teaching. Majority of the women were also married or living with a male partner, with several of these relationships especially in Mpaha, Sankpala and Tidrope being polygynous. The majority of the women also had between 1 and 3 children.

The healthcare providers category of respondents included health professionals (doctors, nurses, midwives, healthcare managers, and health policy-makers or implementers) from health facilities in the study communities, district and regional health directorates, and Ghana Health Service at the national level. The description of the categories of healthcare providers interviewed and their distribution across the study areas are shown in Table 4. These healthcare providers were selected based on their involvement with maternal healthcare at the community, district or national levels.

**Table 4:** Type and distribution of healthcare providers interviewed (N = 20)

Location of healthcare provider	Description of type of healthcare provider	Number of healthcare providers
Ghana Health Service Headquarters (Accra	Directors of policy, planning, monitoring and evaluation, and maternal and child health	2
Ashanti Regional Health Directorate (Kumasi)	Public health director, and public health nurse	2
Northern Regional Health Directorate (Tamale)	Public health director, and public health nurse	2
Bosomtwe District Health Directorate (Kuntanase)	Director of health services, and public health nurse	2
Central Gonja District Health Directorate (Buipe)	Director of health services, and midwife	2
Tamale Teaching Hospital	Midwife	1
Kuntanase Hospial	Medical director and midwife	2
Piase Health Centre	Midwife	1
Buipe Rural Clinic	Midwife and medical assistant	2
Sankpala Health Centre	Midwife	1
Mpaha Health Centre	Community health nurses	3

### Sampling and recruitment

For the women, a mix of purposive and convenience sampling techniques was used. The selection was however based on a number of pre-set inclusion criteria: ease of recruitment, participant’s availability, willingness to participate in the study, ability/capacity to consent to participate in the research, and good knowledge or understanding of the interview language. Following consultation with community chiefs and elders, and their subsequent approval of the study, the actual recruitment process involved advertising the study at local churches, mosques, water collection points, and women group meetings in the six study communities via community and religious leaders, women leaders and Community-Based Surveillance Volunteers (CBSVs) (i.e. recruits from local communities who have been trained by the District Health Management Team in various aspects of community health, including but not limited to reporting the outbreak of any disease, and recording births and deaths in their communities). The CBSVs then helped the researchers to recruit interested individual participants for interviewing. Having grown up in the study communities, the CBSVs were very conversant with the local dialect and cultural nuances and were therefore in a good position to advise the researchers on suitable participants as well as arrange interview meetings. In all, 185 women were interviewed.

For all research participants under the ‘healthcare providers’ category, a purposive sampling procedure was used. This was also a judgmental selection of participants based on the researchers’ evaluation of the relevance of their roles or knowledge to the research topic. In total, 20 healthcare providers were interviewed.

### Data collection

Focus group discussions (FGDs) were the main data collection methods. This data collection technique was adopted partly because of its practical relevance in helping to explore how intra-familial decision-making affects women’s access to, and use of, maternity care services in a normal peer-group conversation. Additionally, because FGDs were interactive, participants were able to query and challenge each other as well as explain themselves; hence offering validated data on the extent of consensus or diversity.

In all, 12 focus group discussions – two in each study community - were completed. Women in groups were segmented by age (i.e. 18–30 years, and 31–45 years) because initial discussions with CBSVs suggested that there were age hierarchy conflicts among women in the study communities. In other words, younger women (18-30years) were unlikely to freely express their views in the presence of older women (31-45years) because of cultural norms, which require young people to listen to older people. Segmenting discussants by similar age groups contributed to making participants more confortable when expressing their opinions or sharing their experiences within the group context. To limit the effect of any participant dominating the discussion, all participants were constantly encouraged, especially the quieter ones, to speak, share their opinions as well as agree and disagree with others where they felt the need to do so. Coupled with a mix of directed and non-directed facilitation, the effects of dominant participants on the rest of the other participants’ responses were also significantly minimised. In addition, themes and issues raised and discussed during FGDs were summarised and orally presented to participants to confirm, alter or reject at the end of the discussion. This was to make sure that the information collected accurately represented what participants said.

All focus groups were held in the study communities at venues convenient to both women and the research team. Groups consisted of 9–12 participants. Apart from few children under-five years who accompanied their mothers, no other persons were allowed to sit in the discussions. Discussions in the focus groups lasted 2.5 to 3 hours, and ended when a point of saturation was reached (when no new issues seemed to arise). All discussions were conducted in the local dialects – *Twi* in Kuntanase, Abono and Piase; *Dagbani* in Sankpala and Tidrope; and *Gonja* in Mpaha. Three of the seven-member, all Ghanaian research team (JKG, JYY and VM), conducted all the discussions and interviews. While all members of the research team were fluent in English and at least one of the native languages of the study respondents, the three researchers were fluent in English and all the three native languages of the respondents. None of the research team members however resided in the study communities. There were therefore no known relationships between the researchers and participants.

To complement the focus groups, 44 % (81) of all women who completed the FGDs were purposively reselected and interviewed individually for a more in-depth discussion of some of the points that were raised during the focus groups. The choice of this data collection technique was informed by both the literature and practical considerations. It has been argued that people may be limited in talking about some sensitive but pertinent healthcare experiences in a group context [40]. In several cases, some women in this study declined to tell their reasons for not accessing and using skilled birthing services offered at healthcare facilities in the group. Instead, they suggested that if the researchers wanted to know their reasons, they were happy to discuss those reasons and personal experiences privately with the researchers. For this reason, the focus groups data were triangulated with individual interviews. A major advantage of this method was that it addressed sensitive issues such as personal experiences of childbirth and questions regarding how intra-familial decision-making affect women’s ability to access and use maternity care services. In particular, and compared to discussions in focus groups, many women talked more openly and in greater detail about instances where their husbands and mothers-in-law prevented them from accessing maternal healthcare services. These women felt that openly discussing such matters in the group context could lead to undesirable consequences, including gossips, and possible spousal confrontation and abuse, which may then threaten the stability of their family.

Finally, key informant interviews were conducted with 20 healthcare providers (see Table 4). All interviews with individual women were conducted in *Twi*, *Dagbani,* and *Gonja,* while interviews with healthcare providers were done in English. Interviews lasted between 20 and 30 minutes. The individual and key informant interviews with women and healthcare providers were relatively shorter because each interview focused on the specific maternity care experiences of individual women, and explored very specific questions in relation to intra-familial decision-making and access to skilled care. All discussions and interviews were audio-recorded alongside hand-written field notes.

### Instruments

An open-ended thematic topic guide was designed and used to facilitate the conduct of all focus group discussions. This instrument was modified into two relatively shorter versions, which were used for individual interviews with women and key informant interviews with healthcare providers. These instruments were complemented by a short multi-item demographic questionnaire, which was used to obtain socio-demographic information from all women participants. All the topic guides were designed to ensure that similar themes and questions were covered in each discussion or interview. The instruments however had built-in flexibility that allowed for any pertinent but unexpected issues that arose during the interview process to be further probed. The instruments focused on exploring women’s maternity care needs, experiences of seeking or not seeking care, women’s participation in decision-making related to maternal healthcare services utilisation, women’s mobility and women’s agency at the household, family and community levels. To ensure that the instruments were valid, we pilot-tested them in two of the study communities. During the data collection phase, we also engaged in a continuous review of the questions and interview process. This helped to reframe questions, clarify and use more appropriate or easily understandable concepts as the research progressed.

### Analysis

Following the completion of interviews, the data were analysed using the Attride-Stirling thematic network analysis framework [41]. The Attride-Stirling thematic network analysis framework is a method for conducting thematic analysis of qualitative or textual data, which allows for open and methodical discovery of emergent concepts, themes and relationships. This involved several steps.

The first step involved transcription and reading of transcripts and field notes for overall understanding. During and after qualitative data collection, three language specialists – one each for Twi, Dagbani and Gonja - were contracted to transcribe and translate all audio-recorded interviews into English. The three research team members who conducted the interviews then performed back-to-back translations into English on selected transcripts. The aim here was to verify the accuracy of the translations. Only a few errors were noted and these were corrected before coding. All the transcripts and interview notes were then read and reviewed thoroughly by all members of the research team. Notes were made on hard copies of transcripts. This first step was completed with separate summaries for each transcript outlining the key points participants made. Also a preliminary coding structure and a codebook were agreed upon. Second, all transcripts were then exported into NVivo 9 qualitative data analysis software, where the data was both deductively and inductively coded. Data coding continued until theoretical saturation was reached (i.e. when no new concepts emerged from successive coding of data). Third, the completed code structure was applied to develop and report themes. Themes simply represented some level of patterned response or meaning within the data set [42]. Finally, all the themes identified were collated into a thematic chart to reflect basic themes, organising themes, and global themes in line with the Attride-Stirling’s thematic network analysis framework [41].

To ensure that the thematic chart reflected the data, the data segments related to each theme were thoroughly examined. Where necessary, refinements were made. The final thematic framework constitutes the structure of the findings and discussion sections of this paper. Where appropriate, verbatim quotations from interview transcripts were used to illustrate relevant themes. We followed Tong, Sainbury and Craig’s [43] recommended consolidated criteria for reporting qualitative research (COREQ) to guide our analysis and reporting of findings from this qualitative study.

### Ethics

Ethical clearance was obtained from the University of Oxford Social Sciences and Humanities Inter-divisional Research Ethics Committee (Ref No.: SSD/CUREC1/11-051), and the Ghana Health Service Ethical Review Committee (Protocol ID NO: GHS-ERC 18/11/11). In addition, informed written and verbal consent was obtained from all research participants. Participants did not receive any monetary compensation for participating in the research. However, two participants who travelled to participate in the study were reimbursed for transport. Refreshment with soft drinks and biscuits were also provided after focus group discussions.

## Findings

Participants’ accounts in relation to the influence of intra-familial decision-making power on accessibility to, and utilisation of maternal healthcare services in Ghana centred on four main overarching (global) themes. These are explored below.

### Significance of childbirth and the need for skilled care

We begin the presentation of our results by briefly reporting findings on women’s perspectives on the significance of childbirth, the need for skilled care, and some transitions in relation to women’s preferred place of birth. Discussions and interviews with women and healthcare providers reveal that childbirth is of particular importance to men and women in Ghana. In a matrilineal Ghanaian society, a woman needs to have children to ensure the perpetuity of her own lineage, or that of her husband’s in a patrilineal society.

*It is important for every woman in this community to give birth. If you are a woman and you don’t give birth, then your lineage or even that of your husband will collapse. For us married women, you can’t say you will not give birth, because your husband will just divorce you. This is why I think it is important to give birth, and this is why I am now pregnant* (Pregnant Woman, FGD, Kuntanase).

Because of the value men and women place on childbirth, several of our research participants in both urban and rural contexts emphasised the need for proper care for pregnant women to facilitate safer childbirth. In this regard, there was a general sense of awareness among women about the potential benefits of seeking skilled antenatal care (ANC), delivery care (DC) and postnatal care (PNC) services from a health facility.

*I think it is very important for every pregnant woman to go to hospital to check her pregnancy. I also believe giving birth in the hospital is the best…it can save the lives of the mother and her newborn* (Lactating Mother, FGD, Sankpala).

Indeed, focus group discussions and interviews with women and healthcare providers alike reveal that most women do want skilled assistance in a health facility setting during pregnancy, childbirth, and immediately after childbirth. This is not only because of the importance families place on safer childbirth, but also because of fears that a woman might die in the process of giving birth. Despite this, both women and health providers reported that in practice, women’s limited autonomy in relation to decision-making and physical mobility within the family set-up was one major factor that influenced women’s decision-making regarding whether to seek care or not.

### Women’s autonomy and maternal health-seeking behaviour

The majority of statements women made in focus groups and individual interviews indicate that women face difficulty in accessing or using skilled maternal health services at health facilities because they often lack the independence to make decisions even in situations where they want to seek care. Table 5 presents a count of statements women made in relation to the principal decision-maker(s) in episodes where a woman was unable to access or use maternity care services during her last pregnancy.

**Table 5:** Key household and community decision-makers regarding use and non-use of skilled maternal health services

Who in your household, family, or community made the final decision the last time you were unable to access or use skilled antenatal or delivery or post-delivery care services?	Frequency of statement	Per cent
Husband	88	49.2
Mother-in-law	29	16.2
Husband + mother-in-law	22	12.4
Mother	14	8.1
Traditional birth attendant	10	5.4
Other community members	5	2.7
Self	5	2.7
Mother-in-law + mother	5	2.7
Husband + mother	1	0.5
Total	179	100

Note that the frequencies and percentages reported in Table 5 are not representing the number of women who took part in the study. Rather, they represent a summary count of statements that women made in both FGDs and IDIs when they were asked to indicate the principal decision-maker(s) in episodes where a woman was unable to access or use skilled maternal healthcare services during her last pregnancy. This explains why it is that Table 5 shows a total of 179 statements instead of 185, which is the number of women participants. Indeed, we acknowledge that this quantitative representation of the qualitative accounts women gave is perhaps not a conventional way of presenting qualitative data. Nonetheless, we employ it as a pragmatic technique to facilitate easy communication and understanding of the data.

In nearly half (49.2 %) of the cases as shown in Table 5, the final decision-maker was the husband. In the second highest (16.2 %) number of cases, mothers-in-law made the final decision regarding non-access, while in 12.4 % of the cases husband and mother-in-law were the final arbiters. Strikingly, only in 2.7 % of the cases were women themselves the final decision-makers. In what follows, participants’ accounts on the specific ways husbands, mothers-in-law, mothers, TBAs, and other community members influence their ability or inability to access and use maternal healthcare services are explored.

### Women’s participation in maternal healthcare decision-making

In FGDs and individual interviews, women reported that although they were often expected to nurture their pregnancies and successfully give birth to healthy normal babies, the power to make decisions regarding how and when to seek pregnancy and birthing care is mostly not entirely theirs. Accordingly, such powers are dispersed among a complex network of actors, with husbands and mothers-in-law exercising the greatest share of authority as final decision-makers. One participant illustrates the point:

*The problem is that sometimes it is not our fault that we do not go to hospital to check our pregnancy or deliver in hospital. I say this because when I was pregnant I did not go to hospital until it was 8 months. I wanted to go but my mother-in-law said I was ok. When it was time for me to give birth too, she said I should deliver at home. You know, my mother-in-law said she gave birth to all her children at home and never had problems. Because of her experience, she did not want me to go to hospital too* (Lactating Mother, II, Sankpala).

One participant also said:

*Are you asking me if I went to the hospital for antenatal care? …No…I did not. I wanted to, but…my husband and mother-in-law said I could not leave the farm work and go for antenatal, after all, I was not sick* (Lactating Mother, FGD, Abono).

While majority of the women reported their limited participation in decision-making concerning utilisation of skilled maternal health services, there were important differences. For instance in communities such as Mpaha, Tidrope and Sankpala where Islam is the predominant religion, Islamic religious beliefs and practices appear to exacerbate women’s inability to participate in reproductive health decision-making. One participant said:

*I believe one major reason why some women in this town do not go to deliver in the hospital is because they are powerless. This is because as women, we are expected to be submissive to our husbands. Because of this, most of us women depend on our husbands to make decisions for us. So if my husband makes a decision that I should not go to give birth at the hospital, I have to obey him. This is important because as a Muslim woman both the Koran and Hadith have placed an obligation on me to be obedient and submissive* (Pregnant Woman, FGD, Mpaha).

Participation in decision-making regarding access to skilled care also appears to be much more limited for rural women compared to their urban counterparts.

*In this [rural] community where many of us women depend on our men, there is nothing a woman can do if her husband says don’t go to the hospital. My friends in the city [referring to Tamale, the capital town of the northern region] are different…for them they are doing their own businesses so they can take care of themselves without depending on a man (*Pregnant Woman, II, Tidrope).

One woman also said *‘you see part of the reason why the women are not going to hospital to give birth is because they depend on other people like their husbands or other family and community members to make that decision’* (Lactating Mother, II, Piase). According to this account, in the event that a decision has to be made on whether a pregnant woman should be send to deliver in a health facility, consultation usually starts with the woman and her husband or the father of the unborn child. From there, and depending on the outcome and situation of the pregnancy, mothers-in-law may then be involved. Other persons such as TBAs, brothers, sisters, and other people in the community may also be invited to help with the decision-making. In Tidrope it was reported in FGDs that even a person alien to the community may be invited to help with decision-making if such a person is deemed to have some knowledge in pregnancy and childbirth or commanded some respect and authority in some other unrelated fields. Although majority of participants agreed that interdependence and the communal system of decision-making were valuable and not necessarily bad, they observed that in the context of maternal health the process very often result in delays in seeking care.

Women in polygynous relationships also appear to experience more difficulties in participating in household decision-making regarding access to maternal healthcare. One woman narrated her experience like this:

*The reason why some women are unable to go to hospital to check their pregnancy or even give birth is because of the type of marriage they get into. I am saying this because of my own experience. The time I was pregnant I wanted to go to hospital to give birth but my husband said no. He said that none of his other two wives had given birth in a hospital before. Because of this I had no option than to obey him* (Lactating Mother, FGD, Tidrope).

Indeed, several of the healthcare providers interviewed in this study reported women’s lack of participation in decision-making concerning the use of skilled pregnancy or delivery care services as a major challenge to improving maternal healthcare in Ghana.

*You see part of the reason why our women are not coming to hospital to give birth is because many of them are poor and have little education. They therefore depend on other people like their husbands to make decisions about whether care is sought or not* (Male Healthcare Provider, KII, Kuntanase).

Several of the healthcare providers noted that the situation was particularly worse for women in communities in the northern region including Tidrope, Sankpala and Mpaha, where the kinship system is patrilineal, and women are transferred between patrilines at the time of marriage, and husbands are usually recognised as having authority over their wives.

*One fundamental challenge we face in this with regards to women’s access to skilled care is the culture of the people. You know, patriarchal norms are very strong in many communities; and so unlike many parts of Southern Ghana where because of the matrilineal system, women are better able to own property and make their own decisions, in this region women are really very disadvantaged (Healthcare Provider, KII, Tamale).*

Another healthcare provider said:

*I think one of the biggest reasons is that many of our women are powerless when it comes to decision-making in the household. This is particularly so with childbirth where decision-making may be collective or communal in which case husbands, mothers-in-law, TBAs and other family or community members might actually have more power in the decision-making process than the pregnant woman herself* (Female Healthcare Provider, KII, Sankpala).

A few healthcare providers reported that women who participated in household decision-making were often more likely to influence decisions concerning their reproductive health, including a higher chance of using skilled maternal health services.

*What I have seen over the years is that those women who have education or who are doing their own businesses are usually able to participate more in making decisions about the healthcare of their family. Such women don’t always have problems coming to the hospital for antenatal or for skilled assistance during delivery. This is very clear if you look at the backgrounds of our patients from our health records* (Female Healthcare Provider, KII, Kuntanase).

In both their collective and individual accounts that women and healthcare providers gave, it was clear that many people within the household, family and community influenced women’s ability to seek professional care. Some healthcare providers therefore suggested that husbands, mothers-in-law and the wider community should be targeted with education programmes that seek to encourage women to use skilled care.

*Going forward, I think we should not just target women alone; we must involve their husbands, mothers-in-law and other community members who may have influence on the decision-making processes regarding access to skilled maternal healthcare (Female Healthcare Provider, II, Kumasi)*

### Women’s relative freedom of movement and access

Apart from women’s limited participation in household level decision-making, focus group discussions and interviews with women and healthcare providers also reveal that most women require the permission of a husband or another male to pursue activities outside the home, including attending antenatal clinic or giving birth at a health facility. This was reported to greatly limit women’s ability to access and use skilled maternal health services.

*As a pregnant woman, I cannot on my own say that I am going to hospital to check my pregnancy. I have to get permission from my husband because he is the one who made me pregnant. He is also the one taking care of me. So even if I feel unwell and my man says no I cannot go to the hospital, there is nothing I can do. That is why some of us do not go to hospital to check our pregnancy in this community* (Pregnant Woman, FGD, Tidrope).

Another participant said:

*It is not that I do not want to give birth at the hospital. The problem is that I have to get permission from my family…I mean my husband to travel to the hospital. But you know I have not been permitted to move out of this village since the start of my pregnancy* (Pregnant woman, II, Abono).

Limitations on mobility were particularly pronounced in rural communities where healthcare facilities are lacking and where access to health services often involves travelling considerably longer distances. For instance, in Tidrope and Abono where there were no health facilities at the time of this research, and the distance from any of these communities to the nearest primary healthcare facility was approximately 20 km, several women reported how they were not permitted to travel to access antenatal and delivery care services. One woman recalls her experiences like this:

*I think part of the reason why some of us do not go to hospital is because our husbands and mothers-in-law do not permit us to travel. I am saying this because of my personal experience. When I was pregnant with my first child, my husband and mother-in-law did not permit me to leave the house to visit the clinic to check my pregnancy. My husband would say ‘Do not go to the hospital alone. I will take you there if I have time’. But he was so busy…he never found the time and so I did not go for antenatal check-up* (Lactating Mother, FGD, Mpaha).

But urban and educated women reported greater freedom of mobility than rural and less educated women.

*I can say that part of the problem is because some women especially in rural areas still need permission from their husbands to travel out of their villages. For those of us in towns, it is better…Personally I don’t need permission from my husband to travel. I just need to tell him where I’m going and why and that is all. But for some women who don’t have formal education like I do or who live in the villages, the situation can be very different* (Lactating Mother, FGD, Kuntanase).

Interviews with healthcare providers corroborated many of the experiences women reported above. Many of the accounts healthcare providers gave suggested that a patriarchal ideology, together with low levels of female education and high levels of economic marginalisation among women particularly in rural Ghana, has created a social, economic and political environment in which women are chronically dependent on men.

*I think one of our biggest challenges is the low status of most of our childbearing women…In many communities in Northern Ghana, women are poorer and usually face several disadvantages including lack of education. Of course this situation is changing especially among the youth in urban areas. However many women still require permission from their partners and even mothers-in-law to undertake certain activities both within and outside the home… and this include seeking permission to travel to a clinic or hospital to obtain antenatal care or deliver at a health facility* (Female Healthcare Provider, KII, Buipe).

Another healthcare provider said:

*Some women don’t come to hospital because they are not given permission to leave the home. You know, husbands or even grand mothers …I mean mothers-in-law, sometimes do not allow pregnant women to move out of the home. Sometimes, it is done indirectly…like if the husband says he does not have money to give to the pregnant woman to travel to access care. In such situations, the only thing we can do is keep educating men and community members about the importance of skilled care during pregnancy or delivery. Also, there is the need to educate the girl child and empower women in general so that they can be less dependent on men (Female Healthcare Provider, KII, Piase).*

### Women’s self-efficacy and access

Despite the limitations many women in the study communities face in terms of active participation in intra-familial decision-making, and freedom of mobility, a few women reported their experiences, which demonstrated self-efficacy and positive defiance or at least the potential for defiance.

*When I was first pregnant, I was not going for antenatal care …my mother-in-law did not allow me to go; she always said I was ok. But inside me I felt like I should go. So one day I told my mother-in-law that I must go to see the midwife whether she likes it or not, and I went. I am very happy that I went because the doctor gave me a lot of advice, which helped me to deliver my baby well* (Lactating Mother, FGD, Mpaha).

Another participant said:

*Me, as soon as I became pregnant and my husband was not willing to allow me to go for antenatal check-up, I told him that he must allow me to go. I told him that this is a matter of life and death, and that if he does not let me go to hospital, I will walk out of our marriage. Because of this, my husband had to allow me to deliver my baby in the hospital* (Lactating Mother, II, Kuntanase).

While a few women reported that they defied the decision of their partner or mother-in-law outright in situations where they needed or wanted skilled care but their husbands or mothers-in-law had decided against using health facility services, others said that they found an influential person in the community or a respected relative to plead with their [women] husbands for permission to access care.

*At first, I had problems with my husband and mother-in-law when I wanted to deliver at the hospital. You know, they said I was lazy that was why I did not want to stay at home and suffer and give birth. My mother-in-law even said that I was going to the hospital because there was something I had done wrong…I mean something like an extra-marital affair which I did not want them to know about. Because of all these issues, I had to talk to a respected friend of my husband’s, who then pleaded with my husband and mother-in-law to permit me to give birth at the hospital* (Lactating Mother, FGD, Abono).

Interestingly, most of the women who reported defying the decisions and wishes of their partners or mothers-in-law were those who were relatively younger (20–30 years), had some level of formal education, and were involved in some income-generating economic activity that made them relatively economically independent.

While majority of the women and healthcare providers interviewed noted that women’s agency was very important in facilitating access to and use of skilled care services, a number of them reported that the process could engender intra-familial and spousal conflicts if care is not taken. An interview with one lactating mother from a polygynous marriage at Sankpala illustrates this point. According to this woman, she decided to deliver in the clinic at a time that her husband had travelled. Three days after she delivered, her husband returned from his travel. The husband reportedly became furious, visited her at the clinic, and demanded to take her home. But soon after leaving the clinic, the husband told her that he was sending her back to her parents. His reason was that none of his first two wives has ever delivered in a health facility, and that she was not only weak but also irresponsible for deciding on her own to go and deliver at the clinic. According to this woman, but for the intervention of community elders, the District Director of Health Services and the midwife at the Sankpala health centre, her husband would have divorced her.

## Discussion

In recent decades, the influence of women’s autonomy on their ability to participate in intra-familial decision-making has emerged as important determinant of women’s reproductive and maternal health seeking behaviour, especially in developing countries [15–20]. In Ghana, few studies have however examined how intra-familial decision-making may affect women’s ability to access and use skilled maternal and healthcare services. Based on three aspects of women’s autonomy (participation in intra-familial household decision-making, freedom of movement, and self-efficacy), this qualitative research paper explored women’s decision-making autonomy within the household, family, and community, and how this affects their ability to access and use maternal healthcare services.

Findings from qualitative analysis of the experiences and perspectives of childbearing women and healthcare providers first showed that most women do want skilled care during pregnancy and childbirth, and that the birthplace choice of most women is starting to shift from the home towards healthcare institutions. The transition reported in this study is consistent with one recent study from rural northern Ghana where traditional maternal and child health practices were giving way to more contemporary ones [17]. Factors driving this transition as reported in this study included increasing awareness of the dangers of unskilled homebirth as well as the desire to give birth to normal healthy children. The findings here suggest the need for continuous community-based health education to highlight not only the dangers associated with unskilled care during pregnancy and delivery but also the benefits of skilled attendance during these periods.

Our findings however showed that despite women’s increasing preference for skilled attendance, women’s limited autonomy - as reflected in their limited participation in intra-familial household decision-making, limited freedom of movement, and low self-efficacy – regarding when to access skilled antenatal care (ANC), delivery care (DC) and postnatal care (PNC) services constitutes a grave barrier to access to and use of health facility maternal health services. This supports previous research that observed low antenatal visits and delivery at health institutions in contexts where women’s autonomy was generally low [24–27]. Our study however revealed that the influences of women’s autonomy and intra-familial decision-making on maternal health-seeking differed for different women. For instance, focus groups and interviews with women and healthcare providers in this study revealed that in many instances husbands, mothers-in-law, and TBAs were the principal decision makers, especially when an obstetric emergency set in. Mothers-in-law whose own experiences of pregnancy and childbirth involved limited access to skilled care saw institutional delivery and care to be unnecessary. As such, they discouraged their daughters-in-law from seeking care from trained providers. In Mali, similar findings have been reported [21]. The findings here are also in consonance with other previous studies in Ghana, which showed that husbands, mothers-in-law, grandmothers and compound heads were some of the lead decision-makers on matters of health seeking [17, 29–31]. But our findings particularly illustrated the fact that the power-play between women, their husbands, mothers-in-law and TBAs as reported in this study generally resulted in women either not accessing needed care or reporting to a health facility only when complications have set in. Also, the effect of such unbalanced power relationship between women and husbands or mothers-in-law at the household level and the effect on access to and use of maternal health services was different for women. Women in communities in which Islam was the predominant religion appeared to be more limited in their ability to participate in reproductive health decision-making. Similarly, rural women and women with limited education appeared much more limited in their ability to participate in decision-making regarding access to skilled care; while women in polygynous marriages also reported greater difficulties with participating in household decision-making concerning use of skilled maternal health services. These differences generally suggest the natural complexity of the relationship between intra-familial decision-making and access to skilled care, and also highlight the need to pay attention to context when examining the influences of women’s autonomy on access to maternal health services.

The fact that many women lacked the decision-making independence, freedom of mobility, and self-efficacy to decide whether to access and use maternal health services in the Bosomtwe and Central Gonja districts of Ghana appears to have been framed by two broad contexts, namely gender inequity (i.e. the allocation of *fair* shares, be it in material resources, social resources or political power, rather than gender equality, which suggests allocation of *equal* shares) and the culture of communal decision-making. In relation to the first context, many of the accounts women and healthcare providers gave suggested that a patriarchal ideology, coupled with low levels of female education, high levels of economic marginalisation of women, and traditional interpretations that define women as distinctly submissive, obedient and subordinate to men, has created a social, economic and political environment in which women are often dependent on men. Although some participants acknowledged that the patriarchal ideology, and the low levels of female education and high levels of economic marginalisation among women were fast changing especially in urban settings and among the younger generation, they reported that a combination of machismo, the culture of female submissiveness and women’s economic dependence on men still created an unequal power relationship between men and women in both public and private spheres. In this unequal power relationship, women often ceded their autonomy and decision-making power to men, including decisions concerning access to and use of maternal health services. Thus not only do men at the household level influence women’s maternal healthcare seeking-decisions, but also they wield and exercise considerable power in either permitting or restricting women’s access to, and use of care services. These findings suggest that efforts and strategies that aim to improve women’s economic status and self-efficacy as well as promote gender equity might have potential benefits for women’s access to, and use of maternal health services. As shown in this study and elsewhere [21], women who appeared less economically dependent on their husbands were more likely to actively take part and positively influence household decision-making concerning access to reproductive healthcare services. Of course, the issue of whether women’s enjoyment of equal decision-making rights automatically results in increased access to skilled care and better maternal health outcomes is an area where limited empirical research exists. Further research to explore and compare the relationships between egalitarian and non-egalitarian gender ideologies and maternal health outcomes will shed more light on the open questions raised by the data reported in this paper.

While it is important to improve women’s bargaining power in the household, it is equally important that efforts are made to directly engage men and mothers-in-law in maternal health issues as suggested above by one of the female healthcare providers. Promoting men’s involvement in issues of maternity care could be particularly useful because as shown in this paper and in other studies [17, 29–31], many women still require the permission of a husband or a male partner to pursue activities outside the home, including attending antenatal clinic or giving birth in a healthcare facility. Strategies for involving men could include the provision of male-friendly services and male-friendly maternity clinics for prenatal and delivery care, as well as couple counselling. While adherence to strong patriarchal norms and cultural perceptions about the role of men as bread winners might hinder men’s effective involvement in matters of maternal health [4, 5], programmes that promote male partners’ involvement could be particularly useful in increasing in men an understanding of the relevance of skilled attendance at birth. This could help men to play more supportive roles in the area of maternal healthcare access.

The other context in which women’s maternity care seeking decision-making could be understood relates to the communal nature of decision-making in the study communities. Many aspects of life in the communities in which this study took place are organised around the family, household and the community. Within this frame, people tended to be interdependent, and decision-making on matters of importance such as seeking healthcare for a sick person tended to be communal in nature. It is this sense of interdependence and communal decision-making that seem to explain why some women lose their autonomous decision-making power to other family and community members. That interdependence and communal decision-making framed some women’s inability to access care is clearly in discord with the emphasis on self-rule or independence in discussions around the idea of autonomy. The findings here are however consistent with those of Mumtaz and Sulway [16, 44] in Pakistan and Gupta et al. [18] in Ghana, where the concept of individual autonomy was found to be inconsistent with a reality of a more collectivist community structure in which decision-making concerning women’s reproductive health occurred. As reported by several women in this study, interdependence and communalism are valued and acceptable ways of social organisation and decision-making. Indeed, these have been widely acknowledged and discussed in African philosophical thinking around the famous concept of *uBuntu* [45]. *uBuntu,* variants of which has been found in many African societies, including Ghana, Kenya, South Africa, Tanzania, Angola and the Democratic Republic of Congo [46, 47], albeit not necessarily under the same name, is understood as a moral and an ethical ideal representing collective solidarity whereby the self is perceived primarily in relation to others i.e. a person is a person through other people [48, 49]. Communalism and interdependence are two central core values of *uBuntu* [45]. As illustrated by women’s and healthcare providers’ accounts, communalism and interdependence are still strong binding forces, which frame how decisions, including those concerning maternity care within the household, family and community are taken. This finding suggests the need for further research to interrogate the notion of women’s autonomy for its conceptual adequacy or usefulness as a determinant of women’s reproductive health in contexts such as Ghana. This is particularly important because policies based on conceptualisation of women’s autonomy in individual terms are unlikely to lead to effective action because of the incongruity between notions of individualism inherent in the concept of autonomy with the socially-embedded nature of communal decision-making within which women live [44]. In the meantime, efforts to encourage women to access and use skilled maternal healthcare services should move beyong individual childbearing women to involve community-based health education campaigns that communicate the importance of women delivering their babies with a relatively well-resourced skilled health professional in attendance. Such campaigns should also take into account this communal structure of decision-making and build upon it to both address those aspects of communal decision-making and practices that constrain women’s ability to access needed care and promote maternal healthcare seeking among women. In this regard, community action groups could be formed to lead and reinforce discussions on the acceptability of shifting local social and gender norms and to increase community responsibility for the health of pregnant women. Recent research by Moyer et al. [17] in rural northern Ghana documented a widespread sense of responsibility that community members felt for women to deliver their babies safely. This increased sense of responsibility was reported to drive up utilisation of skilled birthing services. In Mali also, White and colleagues reported the successful deployment of a similar strategy by CARE USA and CARE Mali to tackle prevailing socio-cultural norms that negatively affected maternal health behaviours and outcomes [21].

Finally, our findings revealed that a few women were able to resist and/or overcome the inhibiting attitudes and decisions of their husbands and mothers-in-law as regards access to, and use of skilled maternal healthcare services. These were mostly young women who had attained some level of education. The few examples of self-efficacy demonstrated by educated women highlights not only an important transition but also the potential of education to empower young women to perform improved decision-making roles in the area of maternal healthcare. In this regard, and as suggested by one of the study participants, greater investment in the education of many young girls could potentially empower women to make decisions alone or in consultation with other family members that could have beneficial consequences for their reproductive health and the wellbeing of their children. In doing this, it is important to acknowledge that the few examples of defiance documented in this study do not necessarily demonstrate healthy communication within the family context, and this may lead to poor relationship quality as shown by the experiences of one of the women in Sankpala. As research by Allendorf [50] in Madhya Pradesh, India, has shown, bad or low family relationship quality decreases access to maternal services in the long term.

The findings and recommendations in this paper should however be read against the backdrop of certain limitations. While an attempt was made during the data generation phase of the research to use more direct indicators of women autonomy, we recognise that other dimensions of the concept might not have been captured and that autonomy could change over time. Also, the research reported in this paper was conducted with only 185 women and 20 healthcare providers. While focusing on a small number of participants enabled greater in-depth understanding of how intra-familial decision-making may influence women’s maternal healthcare seeking behaviours, the limitation of generalising the findings to other parts of the country is acknowledged. Another potential limitation is that this study was a cross-sectional study that includes self-reported data. As such it was not possible to verify the relationship between spoken attitudes and resulting behaviour. Also, interviews were conducted in several languages. It is possible that translation errors could have occurred given that the analysis was all done in English. Finally, only women were asked questions about intra-familial decision-making. The perspectives of other family members such as husbands and mothers-in-laws could potentially enrich the data.

The limitations notwithstanding, our findings contain important strengths and lessons that could be drawn upon to inform policies and programmes. In addition to providing important insights into how intra-familial decision-making influences women’s maternity care seeking behaviours in Ghana, the findings demonstrate how efforts to achieve the maternal health related Millennium Development Goals could be undermined by women’s limited participation in intra-familial decision-making, freedom of mobility, and self efficacy or agency. More importantly, this research highlights the fact that the influences of husbands, mothers-in-law, TBAs and other family members on women’s ability to access and use of maternal health services vary from woman to woman, hence the need to pay attention to context when examining the influences of women’s autonomy on access to maternal health services. Finally, and similar to Mumtaz and Sulway [44] and Gupta et al. [18], this study sheds light on the potential conflict between maternal health education and promotion campaigns that target individual childbearing women as autonomous decision-makers, and the reality that these women live in societies where interdependence and communalism are the structures framing most decision-making, including decisions concerning access to and use of skilled maternal health services.

## Conclusion

The findings of the qualitative research reported in this paper contribute to understanding of how intra-familial decision-making affects women’s access to, and use of maternal healthcare services in parts of Ghana. The findings highlight how the goal of improving access to maternal healthcare services can be undermined by women’s lack of decision-making autonomy through complex processes of gender inequality, economic marginalisation, communal decision-making and social power. The findings suggest that individual childbearing women alone do not make decisions regarding access to and use of maternal healthcare services. Rather, the values and opinions of other family and community members as well as the broader socio-economic context do play influential roles. This suggests a need for interventions targeting a range of actors and stakeholders at multiple levels in the community.
